# Self-rectifying performance in the sandwiched structure of Ag/In-Ga-Zn-O/Pt bipolar resistive switching memory

**DOI:** 10.1186/1556-276X-9-548

**Published:** 2014-10-02

**Authors:** Xiaobing Yan, Hua Hao, Yingfang Chen, Shoushan Shi, Erpeng Zhang, Jianzhong Lou, Baoting Liu

**Affiliations:** 1 College of Electron and Information Engineering, Hebei University, Baoding 071002, People’s Republic of China; 2 The Laboratory of Nano-Fabrication and Novel Devices Integrated Technology, Institute of Microelectronics, Chinese Academy of Sciences, Beijing 100029, People’s Republic of China; 3 Department of Physics, Hebei University, Baoding 071002, People’s Republic of China

**Keywords:** Resistive switching, Self-rectifying, Schottky barrier

## Abstract

We reported that the resistive switching of Ag/In-Ga-Zn-O/Pt cells exhibited self-rectifying performance at low-resistance state (LRS). The self-rectifying behavior with reliability was dynamic at elevated temperature from 303 to 393 K. The Schottky barrier originated from the interface between Ag electrode and In-Ga-Zn-O films, identified by replacing Ag electrode with Cu and Ti metals. The reverse current at 1.2 V of LRS is strongly suppressed and more than three orders of magnitude lower than the forward current. The Schottky barrier height was calculated as approximately 0.32 eV, and the electron injection process and resistive switching mechanism were discussed.

## Background

**Figure 1:**
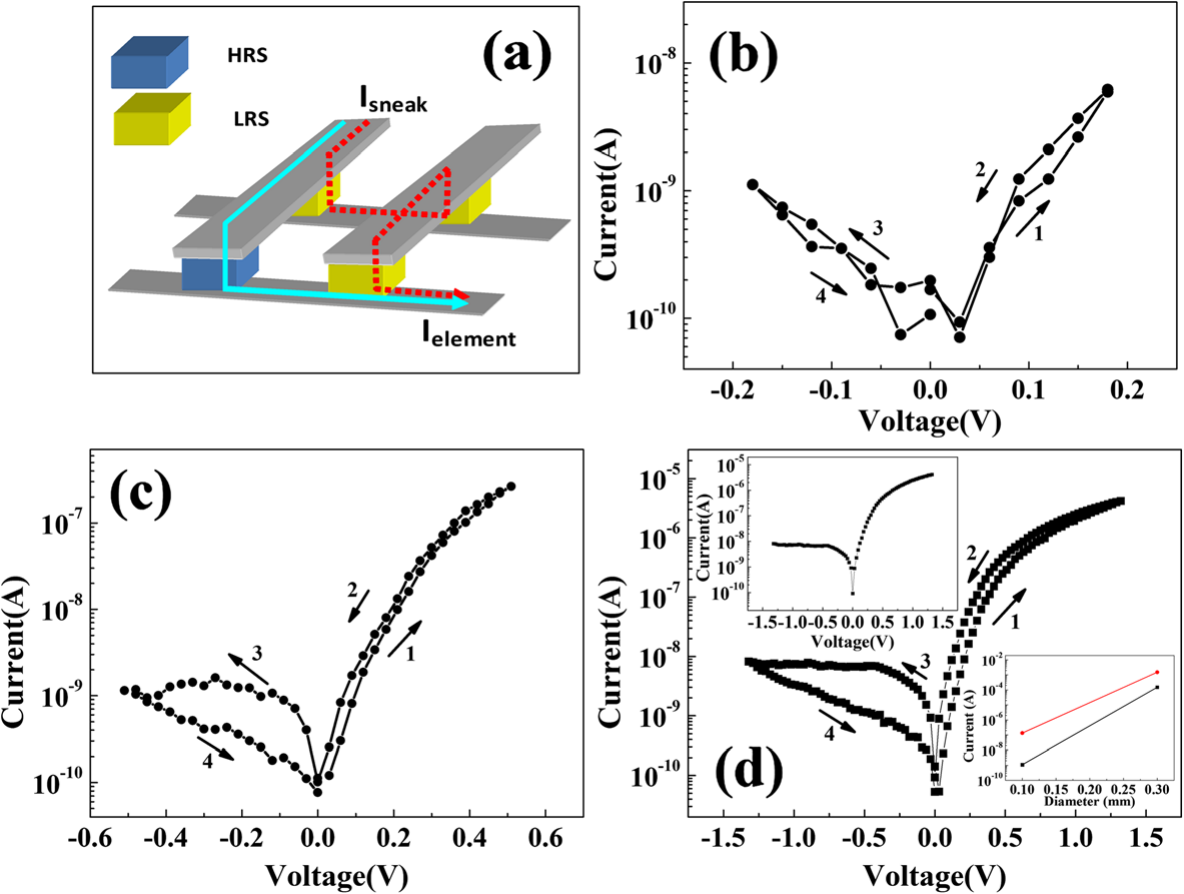
**Schematic of a sneak current path and *I-V *characteristics of Ag/In-Ga-Zn-O/Pt resistive memory. (a)** The sneak current path as a dotted line in cross-bar array. When HRS cell is read, the sneak current path is formed due to the surrounding LRS cells. **(b)**-**(d)** The *I-V* characteristics of Ag/In-Ga-Zn-O/Pt resistive memory on a semilogarithmic scale with different range of voltage sweep, and the voltage is swept in the direction as follows: 1 → 2 → 3 → 4 process. The inset of **(d)** shows the LRS of the resistive memory (upper) and the resistance of the device scaling with the area size of the top electrode (lower).

Resistive switching random access memory (RRAM) has attracted considerable interests for its potential in the next-generation high-density nonvolatile memories due to its low power operation, long date retention, and fast write/read speed [1]. In order to realize high-density memory, the cross-bar architecture, in which memory cells are integrated between the word and bit lines, has been presented for achieving a cell size of *4 F*^*2*^ of RRAM application [2]. However, there is a serious problem for this specific application because of the sneaking current issue due to cross-talk effect in cross-bar structure. For example, in a simple 2 × 2 array, a cell at high-resistance state (HRS) will be misread when three surrounding cells at low-resistance state (LRS) form an unexpected sneak current path as shown in Figure 1a [3]. While in a large memory array, e.g., *m* × *n* (*m*, *n* > 2), the number of the sneak current path is huge, which makes such leakage current unreasonable. Some solutions have been reported in the form of devices such as transistors, selection devices, and complementary resistive switch (CRS) where the logic bit is coded in two different reset (high-resistance) states for RRAM cross-bar integration [3-6]. Especially, a rectifying diode (1D) or transistor (1 T) integrated with RRAM cell is one significant method to block sneak current path [7]. Unfortunately, it will increase fabrication cost and power dissipation for integrating a diode and transistor, which also influences memory performance. Recently, the self-rectifying resistive memory has been actively demonstrated [8,9], which exhibits the obvious rectification effect in LRS, so this manifestation can alleviate the cross-talk effect without serially connecting a diode. Therefore, much work has being done to investigate self-rectifying effect in RRAM. However, it is quite difficult to obtain the self-rectifying characteristics for most the binary metal oxide material, which may be resulted from the fact that conductive filaments (CFs) with metallic property are generated and it leads to an ohmic contact at the interface between the metal electrode and oxide films, so no rectifying behavior was observed. Sawa and Yang et al. reported that complex crystalline oxides Pr_0.7_Ca_0.3_MnO_3_ and Cr-doped SrZrO_3_ can realize the resistive switching with self-rectifying effect [10,11]. In this study, we report another complex oxide amorphous In-Ga-Zn-O as the resistive switching storage films, which were widely demonstrated in TFTs and so on, due to its low processing temperature, good transparency, and high mobility [12]. The metals Ag and Pt were used as top and bottom electrodes. The resistive switching device with good endurance has a remarkable self-rectifying effect at LRS. The Schottky barrier behavior between metal Ag and In-Ga-Zn-O films was investigated in detail, and the electron injection process and resistive switching mechanism in the Ag/In-Ga-Zn-O/Pt device structure were also discussed.

## Methods

The 80-nm-thick In-Ga-Zn-O film was under the ambient pressure of 5 × 10^-4^ Pa. The In-Ga-Zn-O films was prepared on a Pt substrate by magnetron sputtering technique at a power of 100 W in 0.5 Pa atmosphere of Ar + O_2_ mixture (Ar/O_2_ flow rate ratio = 50:25) at 450°C. Then postdeposition annealing process was carried out at 450°C in O_2_ ambiance for 30 min. Then, Ag, Ti, Cu, and Pt were deposited as top electrodes by direct current (DC) magnetron sputtering using a metal shadow mask. The top electrode with 70 nm in thickness was deposited at room temperature. The base pressure of the sputtering chamber was below 2 × 10^-4^ Pa, and the working pressure was 3 Pa maintained by a gas mixture of argon. The diameter of the top electrodes was 0.1 mm, and the DC power was 10 W. Keithley 2400 source-measure unit (Keithley Instruments Inc., Cleveland, OH, USA) and probe station were employed to measure the electrical characteristics and switching properties. The bottom electrode Pt was grounded, and voltage sweeps were always applied to the Ag top electrode.

## Results and discussion

Figure 1 demonstrates the representative *I-V* characteristic with 0.18, 0.5, and 1.3 V max voltage sweep range; the rectification effects were observed Figure 1c,d. The direction of the voltage sweep 0 → 1.3 → 0 → -1.3 → 0 V is denoted by the numbered arrows in Figure 1d. A remarkable resistive switching was obtained, and the memory cell can be switched between HRS and LRS reversibly. Moreover, the resistive switching was of bipolar type because a reversal polarity of voltage was applied to the cell for transforming the resistance state. It is worth noting that there is no current jump in HRS or LRS as in Ag/STO and Ag/electrolyte/Pt structure resistive switching devices, in which formation and dissolution of Ag filaments are ascribed for the resistive switching mechanism [13,14]. In addition, the filaments show localization feature; however, we can find obvious size dependence of current of HRS and LRS. The Ag electrodes with two different areas have 0.1 and 0.3 mm diameters. The resistance of the device is scaling with the area size of the top electrode as shown in the inset of Figure 1d. So, no filaments were formed in our device, and the switching mechanism should be different with the phenomenon of electrolytes, and we would discuss the mechanism in following content. The *I-V* curve of the voltage sweep -1.6 → 1.6 V was measured after the cell was switched to LRS in the inset of Figure 1d. We can observe the Schottky-diode-like behavior in Ag/In-Ga-Zn-O/Pt memory cell, and the reverse current at -1.2 V of LRS is about more than three orders of magnitude lower than forward current at 1.2 V due to the remarkable suppression by the barrier as shown in Figure 1d.

**Figure 2:**
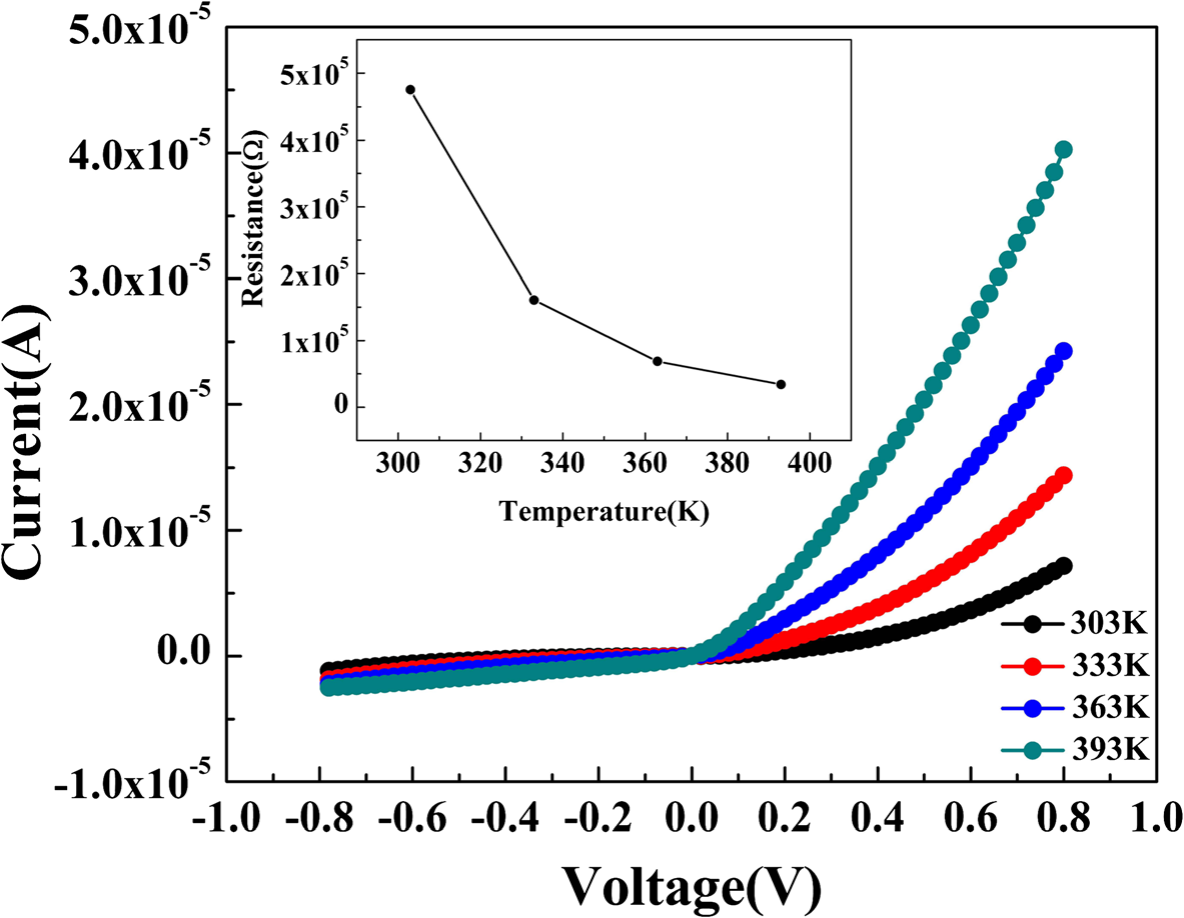
***I-V *characteristics of Ag/In-Ga-Zn-O/Pt in the LRS were tested from 303 to 393 K.** The inset is the temperature dependence of the resistance in LRS.

**Figure 3:**
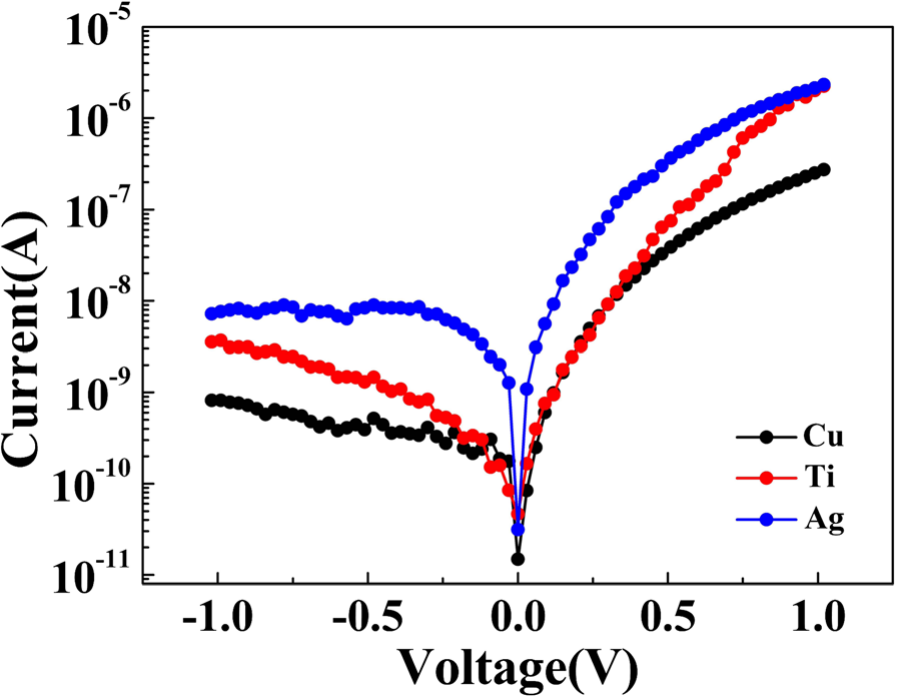
***I-V *characteristics of resistive memory with top Cu, Ti, and Ag electrodes.** The rectifying effect at LRS is shown.

The temperature dependence of the *I-V* characteristics was also investigated for current transport mechanism as shown in Figure 2. The *I-V* curves of the LRS in Ag/In-Ga-Zn-O/Pt were tested from 303 to 393 K. It can be found that the rectifying effects exist at all testing temperature ranges. Besides, the absolute value of the current increases gradually as the temperature increases, implying that the resistance value of LRS decreases as the temperature increases as shown in inset of Figure 2. It could be ascribed to a typical semiconducting behavior, which is similar to the result reported by Chiu et al. [13]. This conducting characteristic in the LRS is notably different from the metallic-filament-based systems [14,15], in which the current in the LRS shows a increasing trend when the ambient temperature increases. Moreover, the rectifying effect is still dynamic at 393 K as shown in Figure 2. In-Ga-Zn-O is an n-type semiconductor, but the devices have larger current in positive segment. The current in the negative part was suppressed. The reverse current is the negative segment; the forward current is the positive segment. Therefore, the self-rectifying effect is due to a potential barrier formation at the Ag/In-Ga-Zn-O interface. To confirm this thought, Cu and Ti are also fabricated as the top electrodes by DC magnetron sputtering. We found that both currents of the negative part of the cell with Cu and Ti electrodes are smaller than the current of the positive part. Compared to the top Ag electrode, a more remarkable rectifying characteristic is observed with top Cu or Ti electrode as shown in Figure 3. This phenomenon indicates that the top electrode dominates the rectifying effect at LRS. It is also natural to suggest that the Ag/In-Ga-Zn-O junction has a Schottky characteristic. Therefore, the barrier formed between the top electrode and In-Ga-Zn-O film is employed to explain the self-rectifying effect. Furthermore, the work functions of Cu, Ti, and Ag are 4.65, 4.33, and 4.26 eV, respectively. Therefore, the higher barriers would be formed at the interface between various top electrode metals and In-Ga-Zn-O films due to the different metal work functions, which are responsible for the different *I*-*V* characteristics. When negative voltage sweep is applied to the top electrode Ag, the barrier at the top Ag electrode/In-Ga-Zn-O interface blocks the electrons flowing from the top Ag electrode to bottom Pt electrode, which makes the negative current much smaller than the positive value. Although the self-rectifying behaviors appear as a dependence on electrode materials with different work functions, it is necessary to investigate the detailed current transport mechanism due to the complexity of the conduction process in the resistive switching device.

**Figure 4:**
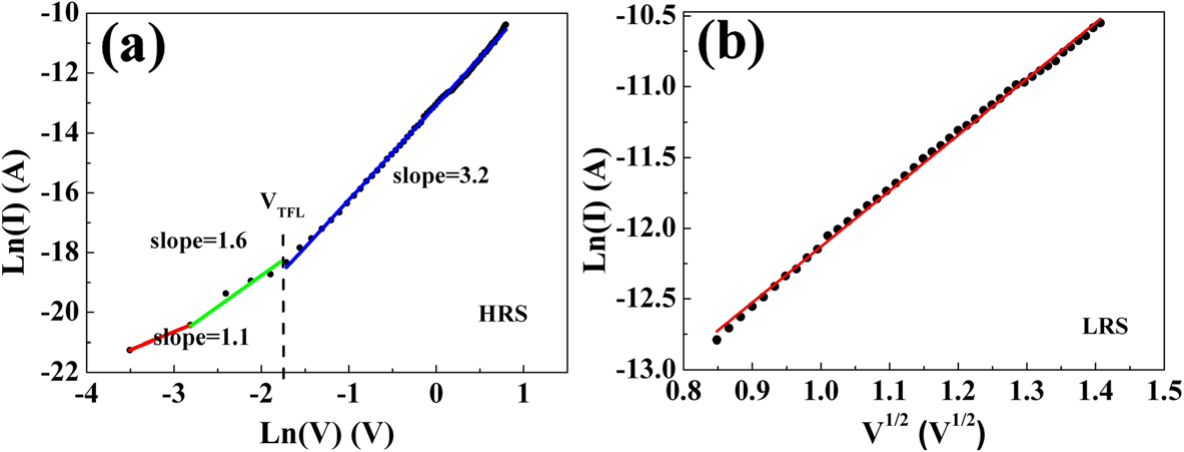
**The ln(*I*)-ln(*V*) curves. (a)** The ln(*I*)-ln(*V*) curves of HRS in the high-field region of Figure 1. **(b)** The ln(*I*) as a function of *V*^1/2^ at the LRS.

To further investigate the resistive switching and current transport mechanisms of the Ag/In-Ga-Zn-O/Pt cell, the *I-V* curve was fitted by the space-charge-limited conduction (SCLC) mechanisms, which is similar to the results reported in [Ref. [16,17]. Figure 4a shows the linear fitting under the relation of ln(*I*) vs. ln(*V*) of the previous *I-V* curve of Figure 1d at the positive voltage sweep region at HRS, respectively. It can be seen from the figure that there are three different slope regions. In region I, the slope is about 1.1, implying the ohmic conduction at the low-voltage region. When the applied voltage is low, there are more thermally generated free electrons than injected electrons from the electrode, thus, the current depends on the applied field and the conductivity of the films. It is followed by a region characterized by a large slope of 1.6, region II, corresponding to Child's law. With the increase of applied voltage, the injected carriers become predominant and a trap filled-limited current is observed, where the *I*-*V* characteristics follow Child's law. In region III, the slope of 3.2 corresponds the accomplishment of trap-filling process [18,19]. The sharp increase of the leakage current occurs at the so-called trap-filled limit voltage (*V*_TFL_) 0.18 V. The injected electrons are transported inside the bulk films (In-Ga-Zn-O) and are partly captured by the traps in the In-Ga-Zn-O films, while others contribute to the total current. This transport process is recognized as trap-controlled SCLC. When the bias voltage is smaller than the trap-filled limit voltage *V*_TFL_ in *I*-*V* curves, the electron injected from the Pt electrode into the insulator In-Ga-Zn-O will distribute in the insulator and cannot transform the insulator to affect the current in In-Ga-Zn-O thin film. So, we also cannot obtain the resistive switching effect as shown in Figure 1b, in which the max voltage sweep is 0.18 V. When the bias voltage exceeds the *V*_TFL_, the electron injected from the bottom electrode Pt can affect the insulator. This can be explained that the deep traps in the In-Ga-Zn-O thin film were completely filled, then the injected electron from the bottom electrode Pt can pass through the thin film, resulting into the increase of the leakage current. In general, it would be caused by the existence of the current jump due to resistance changed by bulk films in set process of their device. As for the *I*-*V* curve of the LRS of Figure 4b, the relation ln(*I*) vs. *V*^1/2^ can be fitted linearly in LRS as shown in Figure 4b, indicating Schottky emission mechanism due to the existence of Schottky barrier at Ag/In-Ga-Zn-O interface. It is also in accordance with above analysis as shown in Figure 3.

**Figure 5:**
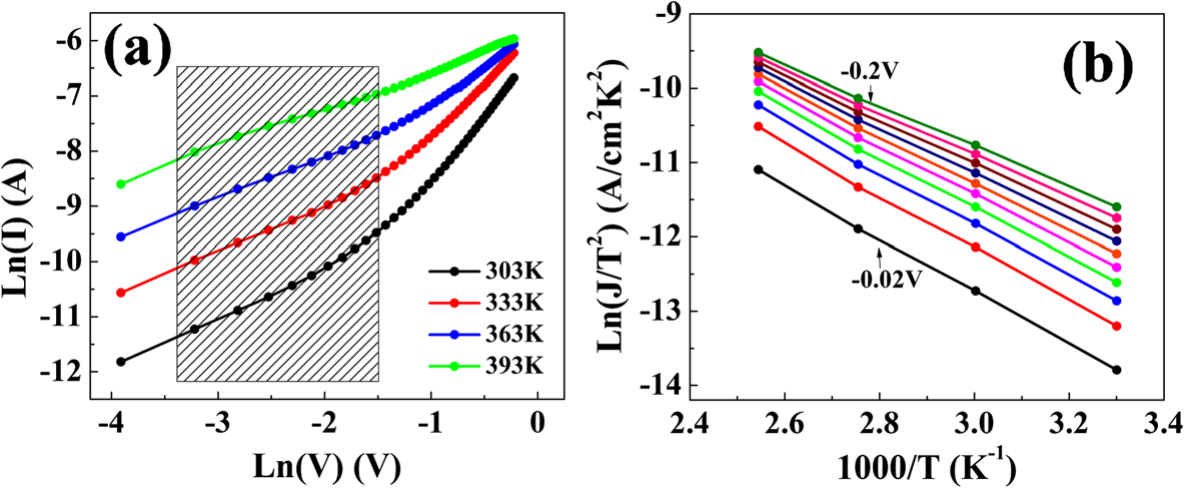
**The ln( *I *)-ln(*V*) curves and conventional Richardson plot of LRS. (a)** The ln(*I*)-ln(*V*) curves of LRS for Ag/In-Ga-Zn-O/Pt resistive memory measured from 303 to 393 K. **(b)** The conventional Richardson plot for the LRS of resistive memory.

In order to confirm the Schottky-type electron injection at the Ag/In-Ga-Zn-O junction of the Schottky barrier, the reverse current of the *I*-*V* curve was measured at temperatures ranging from 303 to 393 K in Figure 5a [20-23]. Figure 5b demonstrates the variation of the Ln(*J*/*T*^2^) at applied voltages ranging from -0.02 to -0.2 V as ranged in shadow area of Figure 5a with the inverse of the temperature according to the Schottky emission in Equation 1:

(1) $$\begin{array}{ll} \;\mathrm{Ln}\left(J/T^{2}\right) & =\left[-q\Phi_{b}+q\sqrt{}\left(\text{qV}/4\pi d\varepsilon_{0}\varepsilon_{r}\right)\right]/\text{kT}\\ & \;+\mathrm{Ln}\left(A^{*}\right) \end{array}$$

where Φ_b_, *d*, *ε*_0_, *ε*_r_, and A* are the interface potential barrier height, depletion layer thickness, dielectric permittivity of the vacuum, dynamic dielectric constant, and effective Richardson's constant, respectively. The activation energies at each voltage $-q\Phi_{b}+q\sqrt{}\left(\text{qV}/4\pi d\varepsilon_{0}\varepsilon_{r}\right)$ were obtained from the slopes. The activation energy decreases linearly with  which is in agreement with the Schottky-type thermionic emission theory. The interfacial potential barriers Φ_b_ at interface were obtained by extrapolating the plots to *V* = 0. By extrapolating to *V* = 0, the Schottky barrier height at 0 V of the Ag/In-Ga-Zn-O interface was determined to be 0.32 eV. The dynamic dielectric constant *ε*_r_ is extracted to be 7.9, according to the fitting results of a Schottky conduction model.

**Figure 6:**
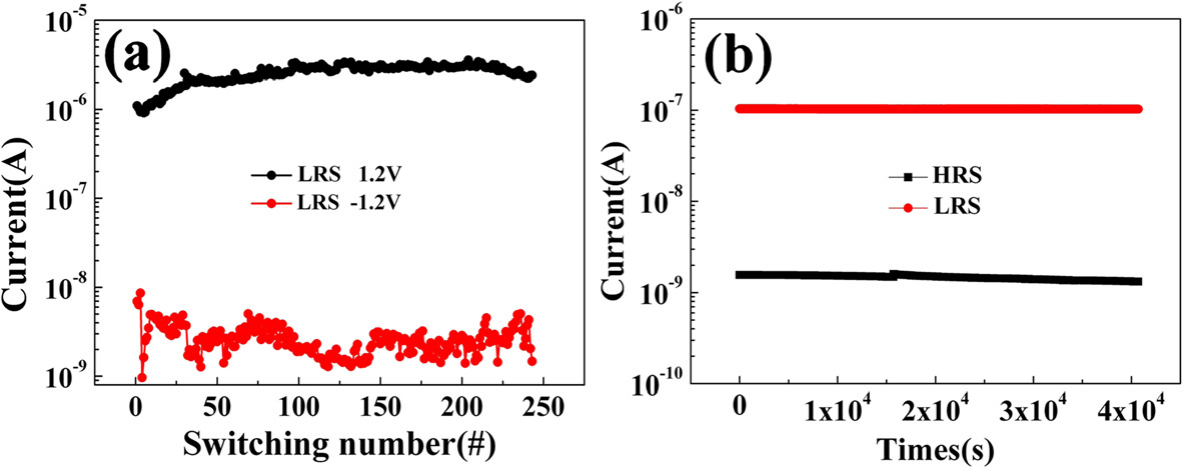
Endurance performance of resistive memory at LRS (a) and retention property of the device (b).

Based on the aforementioned results, the resistive switching device of Ag/In-Ga-Zn-O/Pt with self-rectifying effects shows a potential to be used in cross-bar structure arrays. Therefore, the endurance and retention properties of the cell could be tested as shown in Figure 6a,b. Figure 6a demonstrates the endurance performance of the resistive memory at the LRS. After the resistive memory device was operated for changing to LRS, the current was recorded at -0.5 and 0.5 V as readout voltages, respectively. After 240 cycles of set and reset operation by DC sweeping, the forward/reverse current ratio keeps almost unchanged. Figure 6b shows that the currents of HRS and LRS have no obvious fluctuations after 4.3 × 10^4^ s, implying good retention property. A voltage of 0.25 V was employed as read voltage for HRS for LRS. The reproducible and reliable rectifying effect of Ag/In-Ga-Zn-O/Pt device can effectively reduce the misreading in cross-bar architectures.

## Conclusions

In conclusion, we fabricated the Ag/In-Ga-Zn-O/Pt structure device by RF magnetron sputtering method in this study. The device exhibited good bipolar resistive switching and superior self-rectifying effect. Schottky diode model was employed to explain the mechanism of the self-rectifying characteristics, and the Schottky barrier height is calculated by measuring the *I-V* curves and fitting the data at different temperatures. The experimental results confirm that the resistive switching of Ag/In-Ga-Zn-O/Pt structure can become a promising candidate as non-volatile memory devices using in cross-bar structure.

## Competing interests

The authors declare that they have no competing interests.

## Authors’ contributions

XY conceived of the study, coordinated the research and drafted the manuscript. HH prepared the samples, and YC performed the electrical measurements. All authors, XY, HH, YC, SS, EZ, JL, and BL did the analysis and interpretation of experimental data. All authors read and approved the final manuscript.
